# Supplementary material for: Fungicolous Fungi on Pseudosclerotial Plates and Apothecia of Hymenoscyphus fraxineus and Their Biocontrol Potential
Source: Microorganisms. 2022 Nov 14;10(11):2250. doi: 10.3390/microorganisms10112250 (PMC9697967; doi:10.3390/microorganisms10112250)
Supplement: Supplementary file 1 [file microorganisms-10-02250-s001.zip › microorganisms-1973780-supplementary.pdf]

Table S1. Symptoms on apothecia and pesudosclerotial plates of *Hymenoscyphus fraxineus* up to three weeks after inoculation of ash petioles with test fungi.

Fungal Taxa	Strain Number	Dying of *H. fraxineus* Apothecia [1]		Symptoms on *H. fraxineus* Apothecia	Symptoms on Pseudosclerotium of *H. fraxineus*
		in vivo	in Petrie dishes		
Clonostachys rosea	Fe213F	sc	sc	Initially brown points on the disc, late stage - apothecia turn brown, numerous hyphae and conidia on the hymenium (Figure 5a,b)	Mycelium scarce
	Fe684F	sc	sc		
	Fm884F	sc			
	Fe886F	mn	sc		
	Fe887F		sc		
Clonostachys solani	Fe26K		mn	Initially brown points on the disc, late stage - apothecia turn gray-brown, abundant sporulation on the hymenium (Figure 5c)	Delicate and scarce mycelium
	Fe888F		sc		
Corticiales sp. 1	Fe18K	sc	mn	Apothecia turn gray-brown, numerous hyphae on the stipe and on the receptacle (Figure 5f)	Mycelium scarce, more dense in places
Cordyceps sp. 1	Fe446K	sc	mn	Initially, hyphae are scarce, dying apothecia wrinkled, covered with numerous hyphae and conidia, thin mycelial cords (chemotropism) between apothecia (Figure 5d,e)	Hyphae more numerous only in the vicinity of the apothecia
	Fe457K	sc	mn		
	Fe842F	mn	mn		
Cyathus olla	Fe141F	mn	vn	Apothecia are covered with dense mycelium and decline (Figure 5gh)	Mycelium very abundant, covers the entire petiole
Efibula sp.1	Fe287F	mn	vn	Apothecia covered with dense string-like mycelium and die off (Figure 5i)	Mycelium very abundantly covers the entire petioles, forming mycelial cords
Fusarium sp. 1	Fe167F	sc		Apothecia colonized initially by single thread-like hyphae, the disc of the dying apothecia covered with numerous hyphae; symptoms of chemotropism (Figure 5m)	Hyphae scarce, mainly in the form of single threads
	Fm409E	mn			
	Fm410E		mn		
	Fm507K		sc		
Gymnopus sp. 1	Fe1035F	vn	vn	Apothecia densely entwined by hyphae die off (Figure 5j)	Mycelium very abundant, covers the entire petiole
Minimidochium sp. 1	Fe173F	sc		Apothecia turn uniformly brown, the hymenium is covered with delicate floccose hyphae (Figure 5k)	Hyphae scarce in the form of delicate flocks
Nemania diffusa	Fe831E	mn	sc	Affected apothecia turn brown, in time covered with numerous hyphae (Figure 5l)	Mycelium quite dense, covers quite large fragments of the pseudosclerotium
Pestalotiopsis sp. 1	Fe447K	sc		Apothecia, including hymenium, slowly turn brown, scarce hyphae, threadlike (Figure 5n)	Hyphae scarce, threadlike
	Fe448K		sc		
Polyporales sp. 1	Fe302F	mn	vn	Apothecia, covered with a dense	Mycelium very

	Fe1011F	mn	vn	string of mycelium, die off (Figure 5o)	abundant, covers the entire pseudosclerotium
	Fe1037F		vn		
Trichoderma atroviride	Fe57K	sc	mn	Apothecia turn brown, covered with sparse mycelium and conidia (Figure 5p); new apothecia distorted on some petioles (Figure 5q)	Mycelium cobweb or threadlike, not very profuse
	Fe377E	sc	mn		
	Fe464K	sc			
Trichoderma harzianum	Fe412E		vn	Apothecia die off, abundant accumulation of hyphae on the hymenium (Figure 5r)	Mycelium more numerous mainly between the apothecia
	Fm506K	mn			
Trichoderma polysporum	Fe166F	mn		Affected apothecia die, small aggregates of mycelium on the hymenium (Figure 5s)	Mycelium delicate all over petioles, and local cushion-shaped mycelium clusters
Trichoderma rodmanii	Fe56K	mn		Apothecia infested by a few threadlike hyphae slowly turn brown and die	Hyphae scarce on the entire petiole
Trichoderma tomentosum	Fe460K		mn	The disc affected by the filamentous hyphae turns brown, the apothecia die off	Numerous filamentous hyphae all over pseudosclerotium
Trichoderma sp. 1	Fe242F		mn	Apothecia die relatively quickly, covered with numerous threadlike hyphae (Figure 5t)	Numerous threadlike hyphae in some places
	Fe289F	mn			
	Fe462K		mn		

[1] Dying of *H. fraxineus apothecia*: sc – scarce <10%), mn – moderately numerous (11-50%), vn – very numerous (>50%).
